# Evaluating the impact of task-sequencing on cognitive and motor performance in MS: PASAT and 3-minute walk test order effects

**DOI:** 10.3389/fneur.2025.1654656

**Published:** 2025-09-30

**Authors:** Valentin Siegert, Laura J. Köhler, Lucas Schreff, Daniel Hamacher, Patrick Oschmann, Veit Rothhammer, Philipp M. Keune, Roy Müller

**Affiliations:** ^1^Klinikum Bayreuth GmbH, Bayreuth, Germany; ^2^Universitätsklinikum Erlangen, Erlangen, Germany; ^3^Friedrich-Alexander-Universität Erlangen-Nürnberg (FAU), Erlangen, Germany; ^4^Friedrich Schiller University Jena, Jena, Germany; ^5^Otto Friedrich University Bamberg, Bamberg, Germany; ^6^University of Bayreuth, Bayreuth, Germany

**Keywords:** multiple sclerosis (MS), 25-foot walk, inertial sensors, cognition, Paced Auditory Serial Addition Test (PASAT)

## Abstract

**Introduction:**

Cognitive-motor functioning in persons with Multiple Sclerosis (PwMS) may be studied effectively by means of dual-task paradigms, under which potential impairments may become more salient. However, the influence of task sequencing, i.e., the order in which a cognitive or motor task is administered prior to the dual-task condition remains unclear. This study aimed to investigate potential task-sequencing effects, as reflected in fatigue or learning effects across single- and dual-task conditions.

**Methods:**

A total of 152 PwMS with an average EDSS of 2.3 were quasi-randomly assigned to six groups. The groups differed in the sequence in which a single-motor-task (3-min-25-foot-walk) and a single-cognitive-task (Paced Auditory Serial Addition Test, PASAT) as well as a dual-task combining both were administered. Gait parameters were measured using an IMU sensor. Statistical analyses compared single- and dual-task performance depending on task-sequencing.

**Results:**

Task-sequencing did not affect cognitive or motor performance during the dual-task condition. However, a significant improvement in PASAT scores was observed between the first and second single-task trials, indicating a learning effect. No significant fatigue effects were found in gait parameters between repeated single-task trials.

**Discussion:**

The findings suggest that the sequence of task administration does not significantly influence dual-task performance in the subgroup of PwMS focused on in the current work. Merely repeated single-task use of the PASAT leads to cognitive performance improvements, likely due to learning effects. These results indicate that, in clinical settings, test order may be of minor importance for dual-task conditions, if administered according to the procedure used in the current work.

## 1 Introduction

Multiple sclerosis (MS) is a chronic autoinflammatory disease of the central nervous system. The autoimmune process targets the myelin sheath of neurons in the cerebrum and spinal cord. This process leads to secondary axonal damage and destruction. Persons with MS (PwMS) often exhibit impaired cognitive and/or motor functioning, manifested on the one hand in impaired attention, processing speed (1), executive function and episodic memory [e.g., (2–5)] and on the other hand manifested in gait disturbances, motor fatigue and balance impairments [e.g., (6–9)].

The described cognitive and motor impairments are of great importance for PwMS, particularly in everyday life (e.g., having a walk and holding a conversation concurrently). Everyday situations often involve combinations of motor and cognitive tasks, so-called dual-tasking. In such scenarios, the brain is challenged by two distinct processes: motor skills and cognitive functions. Several studies have been conducted to objectify these dual-task processes in PwMS. The extant research demonstrates that MS is frequently associated with a loss of motor and cognitive performance under dual-task condition [e.g., (10–16)].

In PwMS, performance of the locomotor function is reduced in the dual-task condition (i.e., reduction in step length and walking speed) compared to single-tasking [e.g., (17–19)]. With regard to cognition, the extant literature demonstrates a slight decrease in performance under dual-task conditions (19), which is particularly evident in more complex, commonly administered test procedures (e.g., fixed digit span) (20). The discrepancy in the selection of cognitive [e.g., PASAT (15, 21), serial 7 subtractions (22), count backward by three (23)] and motor tests [e.g., 3-minute-walk test (15), timed up and got test (24), timed 25-foot-walk (16)], as well as the structural and methodological disparities inherent in dual-task studies, nevertheless has the potential to yield methodological confounds. For instance, learning effects related to cognitive tests or fatigue related to endurance in motor tests can affect study outcomes. Learning effects occur when certain tests are carried out several times. The majority of studies utilize single-tasks and subsequently compare these with the results of dual-task components (25–27). The issue of double testing, especially in cognitive tests, is that subjects may remember solutions or develop a strategy for processing them. This implies that statistical outcomes may be contingent on the sequence in which the tests are administered to a certain extent. Consequently, it appears important to explore whether task-sequencing, i.e., the order in which the dual-task versus single-task conditions are administered may be an influential factor in this regard.

In contrast to the learning effects, there is the phenomenon of fatigue (6, 28), a factor that can become particularly evident in PwMS (9, 29–31). When a motor test is repeated under single- and dual-task conditions, fatigue may influence the degree of measured motor impairment, particularly on the last test performed. For example, if the last test performed was under dual-task conditions, a decline in motor performance may not be exclusively attributable to dual-task costs. A proportion of the loss in performance could also be attributable to the presence of fatigue symptoms. The duration of the test is a salient factor in this regard. Studies employing comparatively brief tests (e.g., timed-25-foot-walk) (16) that endure only a few seconds are less prone to induce fatigue. However, there are test models that utilize substantially longer periods (e.g., 25-foot walk repeated continuously for 3 min) (15), and through the implementation of repeated testing between single- and dual-tasks, this may yield a total walking time of up to 6 mins. Hence, also with regard to fatigue symptoms in PwMS (6, 28), it may therefore be assumed that the order of task conditions could have an influence on fatigue and thus confound the results in dual-task experiments.

To the best of our knowledge, there is no general consensus in the concurrent literature concerning the order of tests in dual-task paradigms, and detailed information on the influence of factors such as fatigue and learning effects is scarce (15). The impact of task-sequencing on cognitive and motor performance, fatigue and learning effects in dual-task experiments remains to be addressed in detail. Thus, the objective of this study was to examine the impact of the sequence of administration of a cognitive test [Paced Auditory Serial Addition Test, PASAT (32)] and a motor task [25-foot walk repeated continuously for 3 min (15)] on dual-task performance, as well as on repeated single-task performance. The latter was examined to gain information about potential learning effects and fatigue.

## 2 Methods

### 2.1 Participants

**Figure 1:**
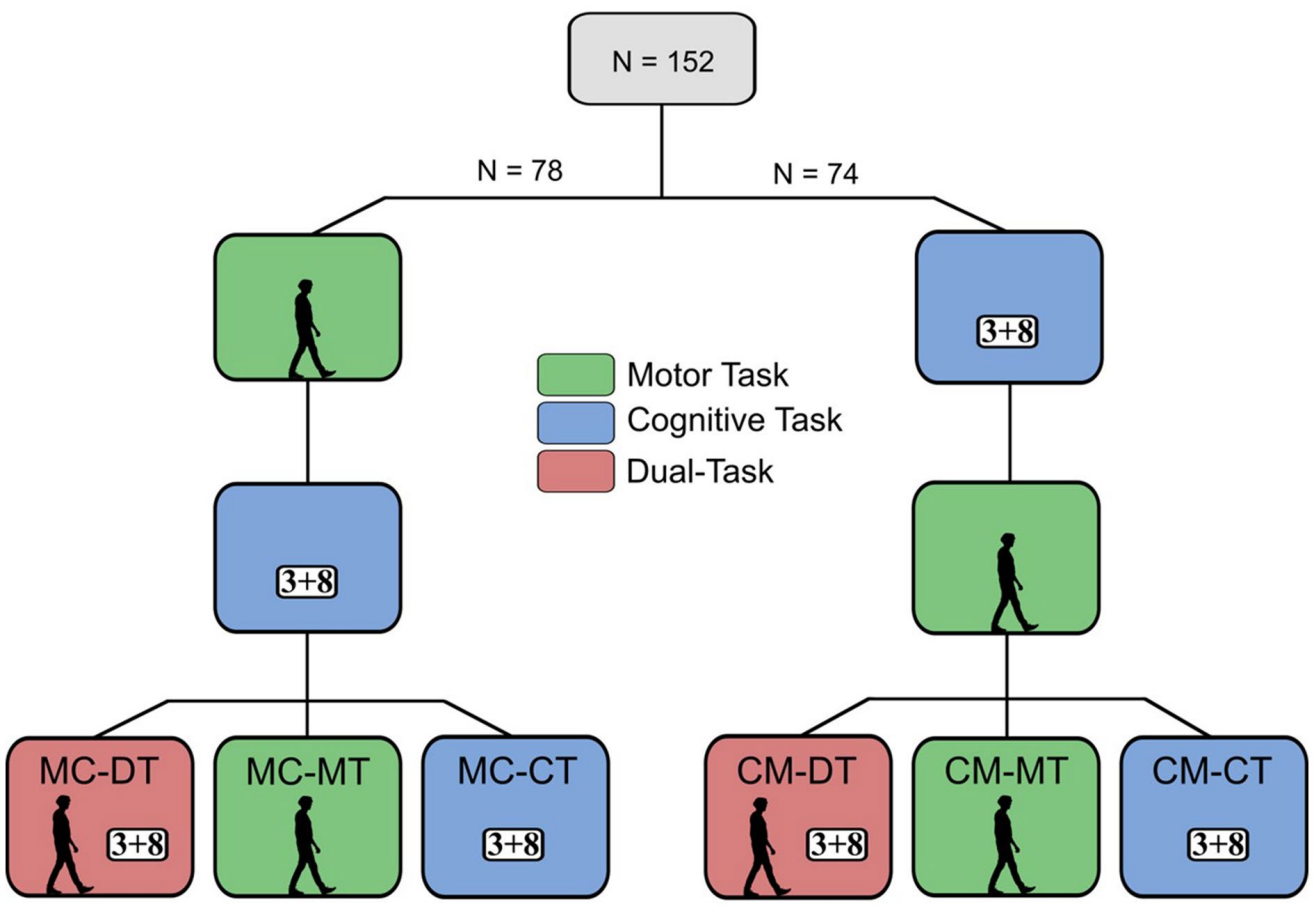
Study design and trial conditions. The walking human silhouette (green box) represents conditions in which the Motor Task (3 min-25-foot walk) was performed, while “3 + 8” (blue box) symbolizes a sample arithmetic task used in conditions involving the Cognitive Task (PASAT). The red box illustrates the dual-task condition.

The sample size was estimated for an ANOVA model (repeated-measures, between factors) conducted by means of G*Power 3.1.9.4 software. This *a priori* power analysis revealed the necessity of 144 participants, given the following input parameters: effect size: *F* = 0.25; alpha error probability: 0.05; power: 0.8; number of groups: 6; measurements: 3 (see Figure 1). Due to the high standard in patient treatment and management, we do not expect the drop-out rate in the current project to exceed 10%, we hence decided to recruit a total sample of 158 participants. All participants were recruited in the Department of Neurology, Klinikum Bayreuth GmbH, Medical Campus Upper Frankonia, Germany. Out of the 158 PwMS, the data of six participants were excluded due to incorrect IMU measurement data. After all a total of 152 PwMS with an average EDSS of 2.3 (Table 1) were included in this study. The following inclusion criteria applied to the study participants: aged 18–65 years, ability to walk for a minimum of 3 mins without the use of assistive devices. Exclusion criteria were hearing impairment, severe cognitive and motor disorders. All PwMS voluntarily participated in the study and provided their written informed consent after they were fully informed about the research protocol, which was approved by the ethical review board of the Friedrich-Alexander-University Erlangen-Nürnberg, Germany (23-202-B) and was in accordance with the Declaration of Helsinki.

**Table 1:** Demographic average data of the individual study groups.

MS group	Age (years)	Weight (kg)	Height (cm)	Gender (m/f)	EDSS
MC-DT (*N* = 26)	44.6 ± 10.5	80.2 ± 14.8	174.7 ± 7.1	10/16	2.1 ± 1.2
MC-MT (*N* = 26)	45.0 ± 11.9	80.52 ± 19.5	168.77 ± 6.8	4/22	2.7 ± 1.5
MC-CT (*N* = 26)	48.4 ± 12.0	78.3 ± 16.2	169.9 ± 9.2	4/22	2.3 ± 1.6
CM-DT (*N* = 24)	47.3 ± 12.9	80.5 ± 17.6	168.7 ± 9.8	5/19	2.1 ± 1.5
CM-MT (*N* = 24)	43.4 ± 13.3	74.2 ± 15.7	169.8 ± 10.1	5/19	1.7 ± 1.5
CM-CT (*N* = 26)	49.6 ± 8.8	76.2 ± 16.8	171.4 ± 9.1	12/14	2.5 ± 1.5
*N* = 152	46.4 ± 11.6	78.4 ± 16.7	170.6 ± 8.8	43/109	2.3 ± 1.5

There were statistically significant differences (shaded area) in height between the MS group MC-DT and the MS group CM-DT, in gender between the MS group MC-CT and the MS group CM-CT, and in EDSS between the MS group MC-MT and the MS group CM-MT (*p* < 0.05). Differences include only group comparisons that were relevant to the study.

### 2.2 Measurements

PwMS were quasi-randomly assigned to six MS subgroups in the order of their recruitment (i.e., the first recruited participant was assigned to group 1, the second recruited participant was assigned to group 2, …, the seventh recruited participant was assigned to group 1, …). The groups differed in the sequence in which single- and dual-tasks were administered (see Figure 1). MS subgroup 1 (MC-DT) commenced with the 3-minute-walk test (Motor Task), subsequently solved the PASAT as a single-task (Cognitive Task), and concluded with the dual-task (DT) consisting of PASAT during the 3-minute-walk test. MS subgroup 2 (MC-MT) and subgroup 3 (MC-CT) initiated similarly to the first trial group and only differed in the final test step. Group MC-MT performed the 3-minute-walk test, while group MC-CT repeated the PASAT. Beginning with MS subgroup 4 (CM-DT), the initial order of PASAT and 3-minute-walk test was reversed (see Figure 1).

#### 2.2.1 Motor test (3-min-25-foot-walk)

The data were collected in the Gait- and Locomotion Lab of the Klinikum Bayreuth GmbH. The motor test was a 25-foot walk which was continuously repeated for 3 min [3-min-25-foot-walk (15)]. To mark the walking distance, two pylons were placed 25 feet apart. PwMS were instructed to continuously walk around a 25-foot course in the shape of an eight, thereby enabling the continuous recording of their gait pattern for a duration of 3 mins. The 3-minute-walk test utilized one IMU sensor (MTw2, Xsens technologies B.V.; angular velocity range ± 1,200 deg./s; frequency 100 Hz) positioned on the dorsum of the dominant foot (6, 33). Walking speed (m/s), stride length (m), stride time (s), stance phase (%), swing phase (%) and minimum toe-to-floor distance (MTC, cm) were determined by means of an established algorithm (34, 35). IMU sensors capture gait parameters adequately compared to a gold standard marker-based camera system (36).

#### 2.2.2 Cognitive test (PASAT)

The PASAT, addressing executive functioning and processing speed, involves the pairwise addition of 60 digits that are auditorily presented at 3-s intervals over a 3-minute period (15, 21, 37). During this process, each time the last-mentioned number is added to the following number, the sum is stated aloud. To ensure that all PwMS comprehended the test setting and, in particular, the PASAT procedure, each participant administered a PASAT practice test, which illustrated the system with 10 sample numbers. The test result was the number of correctly named digits out of a possible 0–60 points.

### 2.3 Statistical analysis

Statistical analysis was performed with SPSS 30 (Chicago, IL, United States). To test normality of distributions, Shapiro–Wilk tests were implemented for all gait parameters (i.e., walking speed, stride length, stride time, the duration of the stance, the duration of the swing phase and minimum toe-to-floor distance) as well as the cognitive performance (PASAT) during single- and dual-task. The homogeneity of variance was tested using Levene’s test. To evaluate the effect of task sequencing (between-subject design), we calculated the “task-costs” (e.g., performance decrement from baseline) for both dual-task (i.e., dual-task-costs = [dual-task – single-task] / single-task * 100%) and single-task (i.e., single-task-costs = [single-task2 – single-task1] / single-task1 * 100%) conditions and performed an independent t-test (i.e., comparing dual-task costs between group MC-DT and group CM-DT, single-(motor)-task costs between group MC-MT and group CM-MT, single-(cognitive)-task costs between group MC-CT and group CM-CT). For the data that were not normally distributed a Mann–Whitney-U-test was assessed. To evaluate the within-subject effect of a cognitive-motor dual-task (i.e., comparing single- and dual-task performance for group MC-DT and group CM-DT), learning effects (i.e., comparing single-task performance for group MC-CT and group CM-CT) and fatigue effects (i.e., comparing single-task performance for group MC-MT and group CM-MT), we performed a paired t-test for normally distributed parameters or a Wilcoxon test for not normally distributed parameters. An alpha level of 0.05 was used for all statistical tests.

## 3 Results

### 3.1 Order effects on dual- and single-task performance

To address the issue of a potential task-order effect on dual-task performance, a comparative analysis was conducted between group MC-DT and group CM-DT. This analysis revealed that there was no significant difference in the dual-task costs of the gait parameters (i.e., walking speed: *p* = 0.909, stride length: *p* = 0.958, stride time: *p* = 0.824, stance phase: 0.755, swing phase: *p* = 0.805 and minimum toe-to-floor distance: *p* = 0.189) or cognitive assessments (*p* = 0.242). With regards to a potential (fatigue) effect on single-task gait performance, the analysis between group MC-MT and group CM-MT revealed no significant differences in single-task costs for all measured gait parameters (i.e., walking speed: *p* = 0.841, stride length: *p* = 0.668, stride time: *p* = 0.776, stance phase: 0.143, swing phase: *p* = 0.177 and minimum toe-to-floor distance: *p* = 0.185). Concerning a potential (learning) effect on the single-task cognitive performance, the analysis between group MC-CT and group CM-CT revealed no significant differences in single-task costs of the PASAT (*p* = 0.229).

### 3.2 Differences between single- and dual-task (dual-task-effect)

Table 2 contains all measured gait parameters and cognitive assessments under single- and dual-task conditions, separately for the MC-DT group and the CM-DT group. For group MC-DT all measured gait parameters changed significantly between the single- and dual-task conditions (Table 2). Specifically, walking speed decreased by 6.9%, stride length decreased by 3.6%, swing phase percentage decreased by 0.5% and minimum toe-to-floor distance decreased by about 8%. Complementarily, stride time increased by 4.6% and stance phase percentage increased by 0.7%. For group CM-DT only four of six measured gait parameters changed significantly between the single- and dual-task conditions (Table 2). More precisely, walking speed decreased by 6.6%, stride length decreased by 3.8%, and swing phase percentage decreased by 0.7%, while stance phase percentage increased by 0.7%. Regardless of the test order, no dual-task effect was observable in case of cognitive performance (Table 2).

**Table 2:** Dual-task results of the different MS groups.

	Single-task	Dual-task	ST vs. DT	DT costs (%)
	MW ± SD	MW ± SD	*p* value	
Cognition (PASAT)				
MC-DT	46.9 ± 12.8	45.8 ± 12.6	0.246	−2.94 ± 17.7
CM-DT	42.4 ± 11.4	42.9 ± 10.5	0.710	3.23 ± 19.1
Walking speed (m/s)				
MC-DT	1.31 ± 0.27	1.22 ± 0.29	0.001	−7.34 ± 6.8
CM-DT	1.22 ± 0.22	1.14 ± 0.27	0.004	−7.03 ± 11.7
Stride length (m)				
MC-DT	1.39 ± 0.19	1.34 ± 0.19	0.001	−3.87 ± 3.7
CM-DT	1.32 ± 0.19	1.27 ± 0.20	0.002	−3.94 ± 5.6
Stride time (s)				
MC-DT	1.08 ± 0.11	1.13 ± 0.17	0.003	4.09 ± 5.9
CM-DT	1.09 ± 0.08	1.14 ± 0.17	0.052	4.68 ± 11.9
Stance phase (%)				
MC-DT	54.6 ± 2.4	55.3 ± 2.7	0.001	1.17 ± 1.3
CM-DT	56.4 ± 2.6	57.1 ± 3.1	0.045	1.01 ± 2.3
Swing phase (%)				
MC-DT	45.2 ± 2.4	44.7 ± 2.8	0.002	−1.34 ± 1.97
CM-DT	43.5 ± 2.7	42.8 ± 3.2	0.027	−1.53 ± 3.3
MTC (cm)				
MC-DT	2.5 ± 0.9	2.3 ± 1.0	0.015	−8.29 ± 17.9
CM-DT	2.4 ± 0.7	2.3 ± 0.7	0.357	−2.15 ± 14.2

All values are expressed as mean (MW) ± standard deviation (SD). ST, single-task; DT, dual-task; MTC, minimum toe-to-floor distance. Dual-task-costs were calculated with [DT-ST]/ST * 100%. Data that are not normally distributed are underlined. Shaded area marks significant differences between ST vs DT.

### 3.3 Differences between single-tasks (fatigue and/or learning effects)

Table 3 contains all measured gait parameters and cognitive assessments under single- and dual-task conditions, separately for the MS groups MC-CT, CM-CT, MC-MT, and CM-MT. Regarding cognitive parameters, PwMS significantly increased their performance. More precisely, in group MC-CT PwMS improved their PASAT result by about 2.4 points and in MS group CM-CT PwMS improved their PASAT result by about 4.3 points (Table 3). Furthermore, gait parameters did not change significantly between both single-tasks neither in group MC-MT nor in group CM-MT (Table 3).

**Table 3:** Single-task results of the different MS groups.

	Single-task 1	Single-task 2	ST1 vs. ST2	ST costs (%)
	MW ± SD	MW ± SD	*p* value	
Cognition (PASAT)				
MC-CT	41.8 ± 11.1	44.2 ± 11.2	0.017	6.58 ± 13.24
CM-CT	42.8 ± 11.9	47.1 ± 11.8	0.001	11.17 ± 13.96
Walking speed (m/s)				
MC-MT	1.24 ± 0.17	1.25 ± 0.17	0.297	0.86 ± 4.1
CM-MT	1.29 ± 0.19	1.30 ± 0.21	0.382	1.18 ± 6.9
Stride length (m)				
MC-MT	1.36 ± 0.14	1.35 ± 0.14	0.784	−0.11 ± 2.4
CM-MT	1.39 ± 0.15	1.39 ± 0.16	0.687	0.25 ± 3.6
Stride time (s)				
MC-MT	1.09 ± 0.08	1.08 ± 0.08	0.061	−0.89 ± 2.3
CM-MT	1.08 ± 0.08	1.07 ± 0.08	0.293	−0.67 ± 3.1
Stance phase (%)				
MC-MT	54.6 ± 1.7	54.4 ± 1.7	0.057	−0.40 ± 0.9
CM-MT	54.5 ± 1.7	54.6 ± 1.8	0.736	0.11 ± 1.5
Swing phase (%)				
MC-MT	45.3 ± 1.7	45.5 ± 1.7	0.060	0.50 ± 1.2
CM-MT	45.4 ± 1.7	45.3 ± 1.9	0.786	−0.10 ± 1.9
MTC (cm)				
MC-MT	2.5 ± 0.7	2.4 ± 0.7	0.625	−0.85 ± 10.9
CM-MT	2.7 ± 0.7	2.6 ± 0.7	0.079	−5.4 ± 13.2

All values are expressed as mean (MW) ± standard deviation (SD). ST1, single-task 1; ST2, single-task 2; MTC, minimum toe-to-floor distance. Single-task-costs were calculated with [ST2-ST1]/ST1 * 100%. Data that are not normally distributed are underlined. Shaded area marks significant differences between ST1 vs ST2.

## 4 Discussion

The present study investigated whether the extent of dual-task effects varied depending on task-sequencing, i.e., the order of a cognitive (PASAT) vs. a motor task (3-minute walk test), administered prior to the dual-task combining both and found that the sequence of the tests does not exert a substantial influence on the outcomes of the dual-task tests. In addition, potential learning effects and effects of fatigue were supposed to be explored by confronting PwMS with repeated single-tasks.

### 4.1 Dual-task order effects

It is noteworthy that there is a substantial body of research on dual-tasks in PwMS [e.g., (14–20, 22)]. However, none of these studies have examined the impact of test sequence. This is surprising and could possibly be explained by the test duration. In studies with comparatively short tests (e.g., 25-foot timed walk), which only last a few seconds, the order might have a smaller effect than tests that utilize substantially longer periods (e.g., 3-min-25-foot-walk). Consequently, we undertook a thorough evaluation of the test sequence, with a focus on discerning any alterations that might have influenced the outcomes. The comparison of group MC-DT and group CM-DT did not reveal any significant differences in the dual-task costs neither in motor nor cognitive performance (Table 2). Hence, in the examined participants, the sequence in which the single-tasks were presented did not exert an influence on the dual-task performance. Participants of the current study were characterized by relatively low EDSS values (average EDSS of 2.3), which reflect relative good walking performance by default (38). A relatively intact gait performance may be regarded as necessary to master consecutive single-task and dual-task walking, as involved in the administered experimental paradigm. Nevertheless, the current results may hence not necessarily be generalized to patients with higher EDSS values. Increased performance deficits are to be expected, especially in PwMS with more severe impairments (22). Therefore it is imperative to investigate the extent to which the test sequence contributes to patient outcomes, particularly in cases of more severe impairments (16).

When looking at the relevant MS subgroups, in group MC-DT we observed a decline in motor performance without any concomitant cognitive changes (Table 1). This finding aligns with the results reported in Kremer et al., who used an identical experimental sequence (15). Compared to group MC-DT, in group CM-DT the test order was reversed (Figure 1). However, similar to the findings observed in group MC-DT, in group CM-DT all measured gait parameters changed in a similar way but differences were only significant in walking speed, stride length, swing phase and stance phase but not in stride time and toe clearance (Table 2). This could be attributed to the small number of participants. While in Kremer et al. 54 PwMS participated in the study, we had only 26 in group MC-DT and 24 in group CM-DT.

### 4.2 Learning vs. fatigue effects

Previous studies have reported dual-task effects, showing a decline in both motor and cognitive performance when tasks are performed simultaneously (10–16). However, in our study motor performance decreased whereas cognitive performance remained constant in both MS subgroups MC-DT and CM-DT when comparing single-task and dual-task conditions. Regarding fatigue, it was hypothesized that motor performance would deteriorate during the second walking trial due to accumulating fatigue effects. However, a comparison of single-task costs between group MC-MT and group CM-MT revealed no meaningful differences (Table 3). This observation suggests that motor fatigue did not significantly influence performance across repeated trials. In contrast, the comparison of cognitive performance between the first and second single-task PASAT trials (group MC-CT and group CM-CT) revealed a notable improvement in test outcomes (Table 3). This enhancement is indicative of learning effects, which are well-documented in previous literature (39, 40). These learning effects must be considered when interpreting the dual-task results. Specifically, while cognitive performance appears stable under dual-task conditions (Table 2), it is likely that this stability is not due to an absence of dual-task interference. Rather, the expected decline in cognitive performance under dual-task conditions may have been masked by the learning effects observed in our study from the previous PASAT experiment. However, the extent to which alternative explanations (e.g., task difficulty) should be considered needs to be investigated in future studies.

### 4.3 Limitations

Some limitations of the present study require consideration. The first limitation is the number of PwMS measured. Despite the 152 PwMS included, it is conceivable that the discrepancies attain significance with an increased number of subjects. For example, stride time increased under dual-task performance in group MC-DT from 1.08 to 1.13 s and in group CM-DT from 1.09 to 1.14 s (Table 2). However, this increase was significant in group MC-DT but not in group CM-DT. Second, despite quasi-randomization demographic differences can be observed between the MS groups (Table 1). For example, there are gender differences between group MC-CT (22 women and four men) and group CM-CT (14 women and 12 men) or differences in EDSS between group MC-MT (average EDSS of 2.7) and group CM-MT (average EDSS of 1.7) that could influence the results of the study. Third, order effects may depend on the test selection. For example, the learning effect may disappear with alternative cognitive tests [e.g., serial 7 subtractions (22), count backward by 3 (23)]. In our case, this could lead not only to a deterioration in motor performance but also in cognitive performance under dual-task conditions. However, the learning effect of these cognitive tests still needs to be investigated in the future.

## 5 Conclusion

The findings of the present study demonstrate that the sequence of the tests does not exert a substantial influence on the outcomes of the dual-task tests, if administered according to the procedure used in the current work. As this procedure, including the PASAT and the 3-minute walk test, has also previously been used successfully (15), it may be recommended as a valid standard dual-task procedure for PwMS with compatible EDSS values. In the context of routine clinical practice, the sequence in which tests are administered, i.e., whether PASAT or the 3-minute walk test precedes the dual-task paradigm, is of negligible consequence.

## Data Availability

The raw data supporting the conclusions of this article will be made available without undue reservation. Requests to access the datasets should be directed to the authors affiliated to Klinikum Bayreuth GmbH.
